# Muscarinic inhibition of salivary glands with glycopyrronium bromide does not reduce the uptake of PSMA-ligands or radioiodine

**DOI:** 10.1186/s13550-021-00770-1

**Published:** 2021-03-12

**Authors:** V. Mohan, N. M. Bruin, M. E. T. Tesselaar, J. P. de Boer, E. Vegt, J. J. M. A. Hendrikx, A. Al-Mamgani, J. B. van de Kamer, J.-J. Sonke, W. V. Vogel

**Affiliations:** 1 Department of Radiation Oncology, The Netherlands Cancer Institute, Plesmanlaan 121, Amsterdam, 1066 CX The Netherlands; 2 Department of Nuclear Medicine, The Netherlands Cancer Institute, Amsterdam, The Netherlands; 3 Department of Medical Oncology, The Netherlands Cancer Institute, Amsterdam, The Netherlands; 4 Department of Radiology and Nuclear Medicine, Erasmus University Medical Center, Rotterdam, The Netherlands; 5 Department of Pharmacy and Pharmacology, The Netherlands Cancer Institute, Amsterdam, The Netherlands

**Keywords:** Salivary glands, Toxicity, PSMA, Iodine, Radionuclide therapy

## Abstract

**Rationale:**

Salivary glands are highly perfused and express the prostate-specific membrane antigen (PSMA) receptor as well as the sodium—iodide symporter. As a consequence, treatment with ^177^Lu/^225^Ac-PSMA for prostate cancer or ^131^I for thyroid cancer leads to a high radiation dose in the salivary glands, and patients can be confronted with persistent xerostomia and reduced quality of life. Salivation can be inhibited using an antimuscarinic pharmaceutical, such as glycopyrronium bromide (GPB), which may also reduce perfusion. The primary objective of this work was to determine if inhibition with GPB could provide a considerable (> 30%) reduction in the accumulation of administered ^123^I or ^68^Ga-PSMA-11 in salivary glands.

**Methods:**

Ten patients who already received a whole-body ^68^Ga-PSMA-11 PET/CT scan for (re)staging of prostate cancer underwent a repeat PET/CT scan with tracer administration at 90 min after intravenous injection of 0.2 mg GPB. Four patients in follow-up after thyroid cancer, who had been treated with one round of ablative ^131^I therapy with curative intent and had no signs of recurrence, received ^123^I planar scintigraphy at 4 h after tracer administration without GPB and a repeated scan at least one week later, with tracer administration at 30 min after intramuscular injection of 0.4 mg GPB. Tracer uptake in the salivary glands was quantified on PET and scintigraphy, respectively, and values with and without GPB were compared.

**Results:**

No significant difference in PSMA uptake in the salivary glands was seen without or with GPB (Mean SUL_mean_ parotid glands control 5.57, intervention 5.72, p = 0.50. Mean SUL_mean_ submandibular glands control 6.25, intervention 5.89, p = 0.12). Three out of 4 patients showed increased ^123^I uptake in the salivary glands after GPB (Mean counts per pixel control 8.60, intervention 11.46).

**Conclusion:**

Muscarinic inhibition of salivation with GPB did not significantly reduce the uptake of PSMA-ligands or radioiodine in salivary glands, and can be dismissed as a potential strategy to reduce toxicity from radionuclide therapies.

## Introduction

### Salivary gland toxicity after radionuclide therapy

Salivary gland toxicity, especially xerostomia, is a serious side effect of several radionuclide therapies (RNTs). The most important examples are radioactive iodine (^131^I) therapy, which is used in the treatment of patients with well-differentiated thyroid cancer [1–4], and prostate-specific membrane antigen (PSMA)-targeted radioligand therapy, for patients with metastatic prostate cancer using [^177^Lu]Lu- or [^225^Ac]Ac-PSMA ligands [5–10]. Both these systemic RNTs target only tissues that express specific proteins; the sodium iodide symporter (NIS) in the case of ^131^I therapy and the PSMA receptor in the case of PSMA therapy. However, the salivary glands (and some other non-target organs) also express these proteins [11, 12], and due to their highly perfused vasculature, also accumulate radiopharmaceuticals targeting these proteins in high concentrations, causing radiation-induced toxicity. The level of PSMA receptor expression in the salivary glands is highly disputed [12–18]. A large fraction of the uptake of PSMA-ligands in the salivary glands is now hypothesised to be non-specific in nature and does not involve the receptor [18, 19].

### Development of strategies to reduce this toxicity

Xerostomia due to the aforementioned RNTs can result in vastly decreased quality of life. Salivary gland toxicity can even be a dose-limiting factor, leading to discontinuation of treatment in PSMA-ligand therapy especially when using alpha emitters [8, 20]. There is a need to find strategies that can reduce uptake preferentially in the salivary glands without affecting uptake in cancerous tissue. Molecular imaging with diagnostic analogues of therapeutic radiopharmaceuticals is often used for pre-therapy dosimetry of tumour and normal tissues, because these diagnostic analogues have identical or almost identical chemical properties as their therapeutic counterparts. For example, ^123^I is chemically identical to ^131^I. Therefore, they are suitable for screening potentially effective strategies to reduce toxicity, by visualising their effect on the tracer biodistribution. Conclusions can then be drawn on organ dose reduction based on changes in diagnostic tracer uptake.

In the case of PSMA-ligand therapy, positron emission tomography with computed tomography (PET/CT) can be used to evaluate the biodistribution of diagnostic radiolabelled PSMA-ligands in vivo [21–23], and can be used to test protective strategies for PSMA-ligand therapies that may reduce uptake in salivary glands. In the case of ^131^I therapy, the uptake in salivary glands can be visualised using ^99m^Tc or ^123^I scintigraphy [24–27], for example to evaluate biodistribution differences after stimulated salivation. The same technique can also be applied to test new protective strategies that aim to reduce tracer uptake.

### GPB to reduce uptake

Glycopyrronium bromide (GPB) is a synthetic anticholinergic/antimuscarinic pharmaceutical that reduces various secretions including gastric, salivary, bronchial and sweat, by competitively inhibiting the cholinergic muscarinic receptors. GPB was mainly used pre-operatively to reduce these secretions, but has also been prescribed to reduce sialorrhea in patients [28]. It significantly reduces the rate of saliva production and can inhibit vasodilation of afferent vasculature via the muscarinic acetylcholine M3 and M1 receptor [29–32]. In contrast to other anticholinergic drugs, it has no effect on the central nervous system due to its inability to pass the blood–brain barrier [33]. We hypothesised that the downregulation of receptors relevant to secretion and vasodilation could also result in lowered perfusion, thereby reducing accumulation of radiopharmaceuticals in the salivary glands. Since GPB’s presumed mode of action is via the cholinergic receptors and not on the NIS or PSMA receptor, it is expected to have no effect on radiopharmaceutical uptake in cancerous tissues. The inhibition of gastric secretion could also lead to less iodine uptake in the stomach, potentially leading to less nausea after therapy.

Among the various strategies tested in the literature, the only one successful in considerably reducing PSMA uptake in salivary glands without affecting tumour uptake is the injection of botulinum toxin [34]. Botulinum toxin, comparable in action to GPB, inhibits the release of acetylcholine and has also been used to treat sialorrhea. However, this technique is spatially limited to injected areas of macroscopic glands, is highly invasive, and could have other potential side effects. We aimed to accomplish the same effects with a systemic mechanism that can reach all gland areas, using a less invasive and potentially less harmful pharmaceutical.

The purpose of this work was to investigate the effect of GPB on the biodistribution of radiolabelled PSMA-ligands and radioiodine. Besides the salivary glands, other tissues were also included in the analysis to study the broader effects of GPB on tracer biodistribution. Kidneys, liver, aorta and malignant lesions were included in the PSMA analysis, and the stomach and intestines were included in the iodine analysis. An uptake reduction of at least 30% in the salivary glands was considered clinically relevant and was selected as the primary endpoint.

## Methods

Both study protocols were approved by the Medical Ethics Committee of the Netherlands Cancer Institute (CCMO trial registration NL65680.031.18 and NL66414.031.18). The studies were conducted in accordance with the Declaration of Helsinki. Written and oral informed consent was obtained from all patients prior to study entry.

### Effect of GPB on PSMA-ligand uptake

#### Study population

The study included 10 patients with prostate cancer, who recently (< 1 month ago) received a [^68^Ga]Ga-PSMA-11 PET/CT scan for evaluation of prostate cancer on clinical indication, and who had at least one visible PSMA positive tumour location > 1 cm in diameter. This first scan served as the baseline scan. Patients who had received any (changes in) treatment since the first scan were excluded. Patients taking anticholinergic medication or having contraindications to such medicine, as well as patients having a history of salivary gland disease or treatment (including radiation therapy to the neck and/or systemic radionuclide treatment) were also excluded.

#### Study procedure and image acquisition

The imaging procedures for the control and intervention scans were identical, except for the administration of GPB. For PSMA PET/CT, patients were first injected with 10 mg of furosemide, and immediately thereafter with 100 MBq of ^68^Ga-PSMA-11 (in-house production according to Dutch legislation and EANM guidelines using local protocols). After an incubation time of 45–60 min, patients were scanned from the upper thighs to the base of the skull using a Gemini TF PET/CT scanner (Philips Medical Systems, The Netherlands). A low dose CT scan was acquired with 150 mAs and 2 mm slices. PET images were acquired with 2–3 min per bed position. All scans were reconstructed iteratively to 4 × 4 × 4 mm^3^ voxels with attenuation correction according to EARL specifications.

The intervention PET scan was performed within a month of the control scan, with the same scanning parameters. On the day of the intervention scan, patients first received 0.2 mg GPB (Robinul®, Biosyn Arzneimittel GmbH, Germany) intravenously, 90 min [33] before injection of furosemide and ^68^Ga-PSMA.

#### Image analysis

Uptake on the PET scans was measured quantitatively using in-house developed software. Standardised uptake values corrected for lean body mass (SUL) were calculated using James’ formula [35]. Large organs with high uptake, namely the salivary glands and kidneys, were segmented using a relative threshold. 3D volumes of interest (VOI) were initially placed around each of the 4 major salivary glands (2 parotid and 2 submandibular glands) and each kidney. A threshold of 20% of the maximum uptake value within each VOI was used to segment them. SUL_max_, SUL_peak_ (defined as the mean of a 1cm^3^ spherical volume centred at the location of SUL_max_) and SUL_mean_ for each of the salivary glands were measured. SUL_mean_ was measured for each kidney. For the liver and aorta, a spherical VOI of 3 cm diameter [36] was placed at the same, representative reference location in both scans, and the SUL_mean_ was measured. For malignant lesions, the lesion with the highest uptake was chosen per patient, and the SUL_max_ was measured. For all paired organs (parotids, submandibulars and kidneys), the mean of the parameters was calculated.

#### Statistical analysis

Statistical analysis was done using IBM SPSS Statistics 25. Comparisons between the control and intervention uptake parameters were done using a two-tailed paired samples t test, after checking for normality according to the Shapiro–Wilk test, assuming a significance level of α = 0.05.

### Effect of GPB on iodine uptake

#### Study population

The study included 4 patients who were in follow-up after primary treatment for differentiated thyroid cancer, who had only received one ablative ^131^I treatment after thyroidectomy, and who had no signs of recurrence at the time of evaluation. The absence of any functional thyroid tissue or tumour recurrence in the neck contributes to straightforward evaluation of uptake in salivary glands, while the maximum of one received ^131^I treatment also minimises possible radiation damage received by the salivary glands. Patients who received new treatments or had changes in medication after the baseline scan were excluded. Patients taking anticholinergic medication or having contraindications to such medicine, as well as patients having a history of salivary gland disease or treatment were also excluded.

The study was initially intended to include 10 patients, with an interim analysis to be carried out after 5 patients were included, to evaluate if the desired trend was exhibited. If the desired trend was not to be found (< 15% uptake reduction in 5 patients), the study would be terminated. Due to difficulties in including the 5^th^ patient (logistical issues and COVID-19), the interim analysis was carried out after 4 patients.

#### Study procedure and image acquisition

The imaging procedures for the control and intervention scans were identical, except for the administration of GPB. After a 4 h fast, patients were orally administered 18.5 MBq (0.5 mCi) ^123^I ([^123^I] Sodium Iodide Capsules, GE Healthcare B.V., The Netherlands). At 4 h post administration of ^123^I, planar images were acquired using a Symbia S eco gamma camera (Siemens Healthcare GmbH, Germany) equipped with a low-energy, high-resolution collimator, with a field of view extending from the top of the skull to the knees. The scan length was 140 cm, acquired at a speed of 6 cm/min. The energy window was centred at 159 keV (145.2–168.8 keV). A standard counting vial of known activity was placed between the patient’s legs. Images were acquired by the anterior and posterior detector panels with a scan matrix of size 1024 × 256, with 2.4 × 2.4 mm^2^ pixels.

One week later, patients returned to receive the intervention scan. For this procedure, patients were administered 0.4 mg of GPB (Robinul®, Biosyn Arzneimittel GmbH, Germany) intramuscularly, 30 min [33] before oral administration of ^123^I.

#### Image analysis

Uptake on the anterior scintigraphy scans was measured quantitatively using in-house software. The posterior scan was not used, and neither was the geometric mean of the two, as this led to a reduction in signal to noise ratio in the regions of interest (ROI). A circular ROI of 3 cm in diameter was centred on the standard vial for every scan. The total counts within the circle were compared between scans to ensure signal consistency. To measure total counts in all salivary glands combined, a rectangular ROI of 60 × 40 pixels (144 × 96 mm^2^) was placed over the salivary glands, ensuring that all activity emanating from the glands fit within it. In order to count the activity from the stomach and intestines, a square ROI of fixed width and length (100 pixels or 240 mm) was drawn ranging from the top of the stomach to the top of the bladder (excluding the bladder). Total counts within the ROIs were measured for the control and intervention scan. The total counts were corrected for decay (time between intake and acquisition) and then divided by the area of the ROI, to get the mean counts per pixel.

#### Statistical analysis

Data were evaluated using descriptive statistics.

## Results

### Effect of GPB on PSMA-ligand uptake

#### Demographics

The 10 male patients had an average age of 72 years (range of 66–82). On the days of the intervention scan, all patients reported the subjective sensation of a dry mouth that began prior to tracer administration and lasted till several hours later. The characteristics of each patient are summarised in Table 1.

**Table 1:** Patient characteristics for PSMA study

Patient	Age (years)	Height (cm)	Weight (kg)	TNM	Initial Gleason score	PSA (ng/ml)
1	68	182	90	T2N1M0	6	13.2
2	76	182	87	T0N1M0 (BCR)	7	1.4
3	82	174	97	T2N0M1	6	14.1
4	66	174	83	T2N0M0	8	7.7
5	75	181	80	T0N1M1 (BCR)	8	2.9
6	74	178	81	T + N0M0 (BCR)	7	3.1
7	64	175	77	T0N0M1 (BCR)	7	8.8
8	74	183	82	T3N0M0	7	19.0
9	72	174	68	T0N1M1 (BCR)	8	1.6
10	73	178	83	T0N0M1 (BCR)	9	0.4

TNM = Clinical stage of prostate cancer, including findings on the baseline PSMA PET/CT; BCR = Biochemical recurrence; PSA = Prostate-specific antigen, measured prior to the baseline PSMA PET/CT

### PET/CT data

**Fig. 1:**
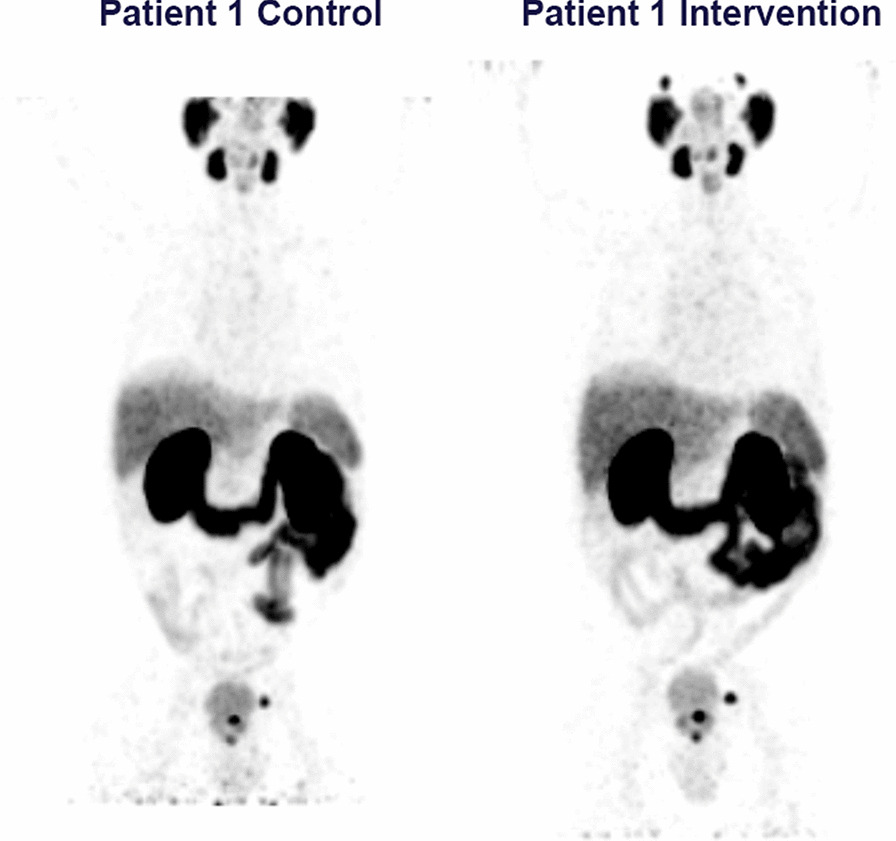
Coronal maximum intensity projections of the control and intervention ^68^Ga-PSMA-11 PET scans of patient 1

Representative control and intervention scans are shown in Fig. 1. The mean ± standard deviation of various SUL parameters for each of the different tissue types investigated in all patients are shown in Table 2. No significant differences between the control and intervention parameters for any tissue type were found, except for a small reduction in uptake in the kidneys.

**Table 2:** Comparison of ^68^Ga-PSMA PET parameters in tissues for control and intervention scans

Tissue	Parameter	Control	Intervention	Relative change (%)	p Value
Parotid Glands	SUL_mean_	5.57 ± 0.93	5.72 ± 1.16	2.7	0.50
	SUL_peak_	10.31 ± 1.46	10.43 ± 1.92	1.2	0.75
	SUL_max_	15.26 ± 2.10	15.28 ± 2.49	0.1	0.97
Submandibular Glands	SUL_mean_	6.25 ± 1.95	5.89 ± 2.21	− 5.8	0.12
	SUL_peak_	10.96 ± 2.37	10.33 ± 2.62	− 5.7	0.10
	SUL_max_	18.82 ± 3.77	17.87 ± 4.57	− 5.0	0.19
Aorta	SUL_mean_	1.08 ± 0.11	1.03 ± 0.13	− 4.0	0.34
Liver	SUL_mean_	4.21 ± 0.65	4.30 ± 0.60	2.1	0.55
Kidneys	SUL_mean_	22.29 ± 5.67	20.31 ± 6.00	− 8.9	0.01*
Tumour or metastasis	SUL_max_	9.71 ± 6.58	9.00 ± 6.62	− 7.3	0.33

SUL = Standardised uptake value corrected for lean body mass corrected; *p < 0.05 is considered statistically significant

### Effect of GPB on iodine uptake

#### Demographics

The 4 patients included had an average age of 51 years (range of 29–67). On the days of the intervention scans, all patients reported the subjective sensation of a dry mouth that began before the time of the tracer administration and lasted till several hours later. The characteristics and ^131^I treatment history of each patient are summarised in Table 3.

**Table 3:** Patient characteristics for iodine study

Patient	Age (years)	Sex	Year of I-131 treatment	Activity (MBq)
1	52	F	1995	2770
2	29	F	2015	3700
3	55	F	2016	1200
4	67	F	2016	1125

#### Scintigraphy data

**Fig. 2:**
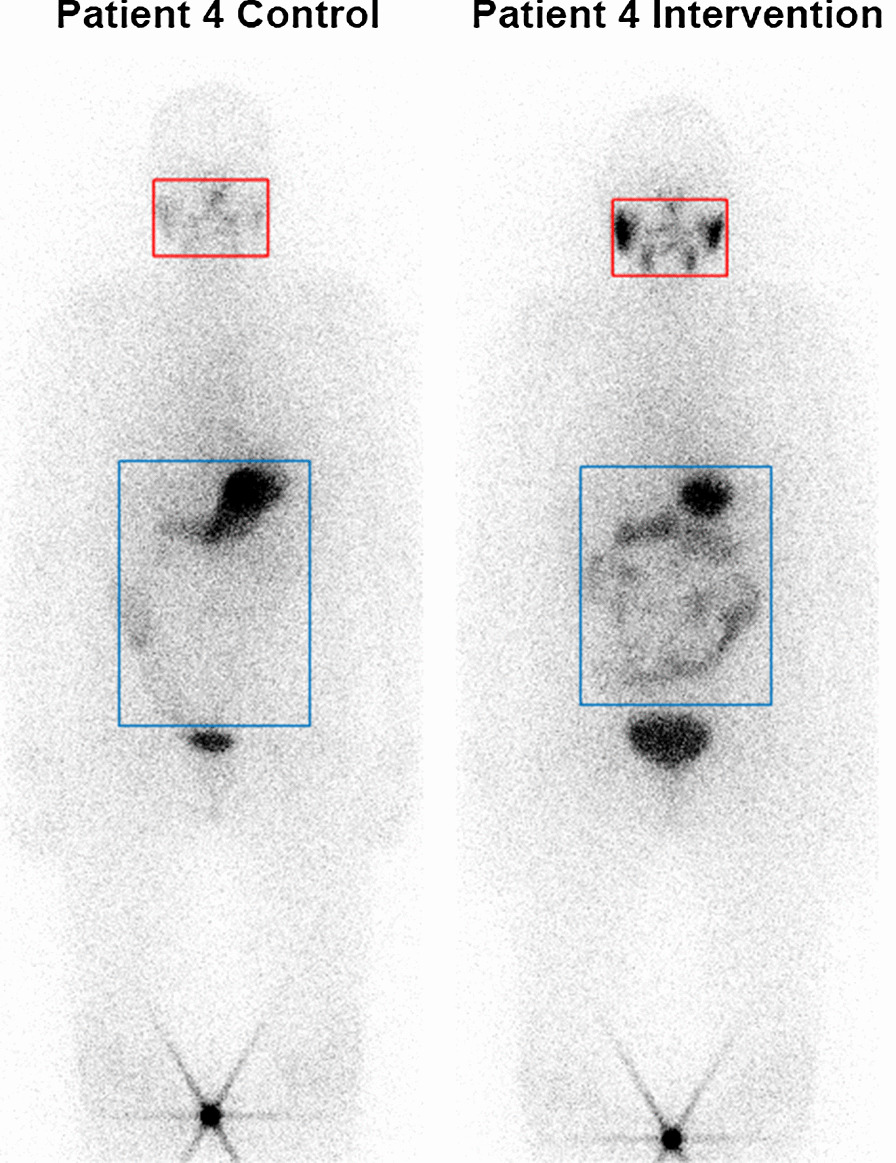
Control and intervention ^123^I planar scans of patient 4. An increase in uptake in the salivary glands is clearly visible on the intervention scan

Representative control and intervention scans can be seen in Fig. 2. The counts within the standard vial did not vary more than 3% between the control and intervention scans for all patients. The mean counts per pixel in the rectangular ROIs for the salivary glands as well as the stomach and intestines are reported in Table 4 for each patient. Averaging the data for the 4 patients, the intervention scans showed a considerable 37% increase in counts in the salivary glands compared to the control scans. Since the opposite effect was observed than was expected, and the desired reduction in uptake was no longer achievable even with the inclusion of a potential 5^th^ patient, the study was terminated for ethical and statistical reasons.

**Table 4:** Uptake of ^123^I without and with premedication with GPB

Patient	Salivary Glands (mean counts per pixel)			Gastrointestinal (mean counts per pixel)		
	Control	Intervention	Change (%)	Control	Intervention	Change (%)
1	7.57	7.74	2.3	7.27	6.95	− 4.5
2	7.10	8.35	17.6	12.76	12.80	0.3
3	11.05	15.84	43.4	13.75	12.42	− 9.7
4	8.66	15.29	76.6	12.04	13.98	16.1
Mean	8.60	11.81	37.4	11.46	11.54	0.7

## Discussion

This work demonstrated that premedication with GPB did not significantly reduce the uptake of PSMA-ligands or radioiodine in the salivary glands, despite successful functional inhibition of the glands resulting in patients complaining of a dry mouth. A limitation of this work is the small number of patients, especially in the iodine cohort. However, it is unlikely that a larger number of patients would yield a reduction in uptake that is clinically relevant, given the trends exhibited.

PSMA-ligand therapies show great promise in treating metastatic prostate cancer. Reducing salivary gland toxicity, especially in the case of alpha emitters, is of paramount importance if the therapy is to transition to wider use in the clinic [7, 8, 20]. The exact mechanism of non-specific PSMA uptake in the salivary glands remains to be elucidated, but its presence indicates that there are a few distinct approaches by which the uptake in the salivary glands could be reduced.

One is by inhibiting the PSMA receptors in the glands, reducing specific uptake. A couple of murine studies [37, 38] evaluated the PSMA inhibitor, 2-(phosphonomethyl)pentanedioic acid (2-PMPA). They found that while its ability to displace renal and potentially salivary gland uptake was substantial, it also resulted in an inhibition of tumour uptake, sacrificing therapeutic efficacy. One study that orally administered polyglutamate folate tablets, a competitive substrate for the PSMA receptor, to patients receiving [^177^Lu]Lu-PSMA therapy, estimated that a reduction in parotid absorbed dose of at least 45% was possible, when compared to other dosimetry studies that did not employ protective strategies [39]. Another recent murine study demonstrated that administering ‘cold’ PSMA could also greatly reduce uptake of [^177^Lu]Lu-PSMA in the salivary glands and kidneys while only marginally reducing tumour uptake [40]. Whether this will translate to human models remains to be seen.

Another method is by reducing non-specific uptake in the glands. Many PSMA-ligands require the integration of a glutamate moiety in order to bind specifically to the PSMA receptor. Recently, a randomised, double blinded, placebo-controlled study attempted to minimise non-specific [^18^F]DCFPyl uptake by orally administering monosodium glutamate, having found favourable results in a previous murine model [41]. In humans however, they found that while the uptake in the salivary glands and kidneys was significantly decreased, so was the uptake in the tumour, once again hampering therapeutic effects [42].

Yet another method is by reducing the perfusion or amount of PSMA delivered to the gland, thereby potentially lowering both specific and non-specific uptake. One study using [^68^Ga]Ga-PSMA PET, tested the hypothesis that applying ice-packs externally, causing vasoconstriction, could reduce perfusion to the glands and thereby reduce PSMA uptake; however, this showed very limited success [43]. This was repeated by another group in a therapeutic setting. Patients who received [^177^Lu]Lu-PSMA therapy with externally applied ice-packs underwent post-therapy SPECT and planar scintigraphy scans, which confirmed that there was no protective effect [44]. Another study investigated the effect of injection of botulinum toxin into the parotid gland and found a significant reduction in [^68^Ga]Ga-PSMA uptake on PET/CT [34]. The result was later attributed to a reduction in non-specific uptake [18, 45].

GPB, however, appears to have no effect on the specific or non-specific uptake of PSMA in the salivary glands. The clinical effect of GPB was apparent, as patients reported feeling a dry mouth for an extended period of time. A possible explanation for the lack of effect on uptake is that GPB, while reducing secretion of saliva, does not affect the delivery of PSMA ligands to the glands. Apparently, the inhibition of salivation and vasodilation does not induce a reduction in resting state perfusion of the salivary glands. PSMA-ligands that are bound to the receptor are not excreted by the glands into the saliva, irrespective of salivation activity. Altogether, the reduction in salivary secretion caused by GPB has no effect on PSMA uptake. We speculate that this suggests the mechanism behind the reduction in PSMA uptake exhibited by the similarly anticholinergic-action of botulinum toxin, is largely specific in nature. The only tissue type that showed a statistically significant difference in uptake was the kidneys. The result was a 9% reduction in uptake on average, suggesting that any nephroprotective effect is quite limited.

Reducing the radiation dose of ^131^I to the salivary glands by using various interventions has been studied before; however these studies have yielded conflicting results [4]. Amifostine, a cytoprotective agent initially thought to be effective in reducing salivary gland toxicity, failed to do so in randomised controlled trials [46]. Pilocarpine, a parasympathomimetic that works preferentially on the salivary glands, also failed to show any protective effects [47]. Various sialagogues, like lemon juice, have also been tested, under the hypothesis that increasing salivation may also increase the secretion of radioiodine, thereby reducing its retention time in the salivary glands. Some studies demonstrated that this resulted in a reduction in uptake [24], while others found an unexpected increase, possibly due to a hypothesised increase in perfusion from stimulation [48, 49]. Our iodine study was conducted after the PSMA study was concluded. Despite the negative findings of the PSMA study, it was decided to test the effects of GPB on iodine uptake, since this is taken up by a different mechanism. The NIS is an active transporter and GPB may downregulate its action.

In our iodine study, all but one patient exhibited a considerable increase in ^123^I accumulation in the salivary glands on the intervention scan. Unlike PSMA, iodine is excreted into the saliva by the glands. The reduction in saliva production caused by GPB, could lead to less of the accumulated ^123^I being excreted by the glands. If the perfusion of the glands and NIS transport are unaffected by GPB, the increase in ^123^I counts in the salivary glands after administration GPB could be explained by this reduced excretion by salivation. Since this effect would also result in more salivary gland toxicity during therapy, we therefore would strongly advise against attempting to use GPB to reduce salivary gland toxicity from ^131^I therapy.

The effect of GPB on the stomach and intestinal uptake of iodine was quantitatively insignificant (< 1%). Visually however, the activity on the intervention scans seemed to ‘leak’ out of the stomach and spread more to the intestines, explicable by a potential reduction in gastric tone which is influenced by cholinergic control [32, 50].

## Conclusion

In conclusion, inhibition of salivation with the antimuscarinic pharmaceutical GPB does not reduce uptake of PSMA-ligands or radioiodine in salivary glands, and therefore cannot be recommended as a means to reduce risk of xerostomia after PSMA ligand or ^131^I radionuclide therapies. Moreover, as demonstrated here, an intervention that may reduce toxicity in one type of RNT might produce the opposite effect for another. Hence, we recommend that protective strategies borrowed from ^131^I therapy be tested first, before applying them on newer PSMA-ligand therapies and vice versa. The search for effective ways to reduce uptake and avoid salivary gland toxicity from RNTs will have to continue.

## Data Availability

The datasets generated and analysed for this work may be available from the corresponding author on reasonable request.

## Abbreviations

^225^Ac: Actinium-225
BCR: Biochemical recurrence
CT: Computed tomography
^68^Ga: Gallium-68 GPB: Glycopyrronium bromide
^123^I: Iodine-123
^131^I: Iodine-131
^177^Lu: Lutetium-177
RNT: Radionuclide therapy
ROI: Region of interest
NIS: Sodium iodide symporter
PET: Positron emission tomography
2-PMPA: 2-(Phosphonomethyl)pentanedioic acid
PSA: Prostate-specific antigen
PSMA: Prostate-specific membrane antigen
SUL: Standardised uptake value corrected for lean body mass
^99m^Tc: Technetium-99 m
VOI: Volume of interest
